# In vitro effects of 6 % hydroxyethyl starch 130/0.42 solution on feline whole blood coagulation measured by rotational thromboelastometry

**DOI:** 10.1186/s12917-016-0767-8

**Published:** 2016-07-26

**Authors:** Nathalie A. Albrecht, Judith Howard, Alan Kovacevic, Katja N. Adamik

**Affiliations:** 1 Division of Emergency and Critical Care, Laenggassstrasse 128, 3012 Bern, Switzerland; 2 Clinical Diagnostic Laboratory, Department of Clinical Veterinary Medicine, Vetsuisse Faculty, University of Bern, Laenggassstrasse 124, 3012 Bern, Switzerland; 3 Division of Internal Medicine/Cardiology, Small Animal Clinic, Department of Clinical Veterinary Medicine, Vetsuisse Faculty, University of Bern, Laenggassstrasse 128, 3012 Bern, Switzerland

**Keywords:** Hydroxyethyl starch, Colloid, Hemostasis, Thromboelastometry, Cat, Coagulation

## Abstract

**Background:**

The artificial colloid, hydroxyethyl starch (HES), is recommended for intravascular volume expansion and colloid-osmotic pressure enhancement in dogs and cats. A well-known side effect of HES solutions in humans and dogs is coagulopathy. However, HES-associated coagulopathy has thus far not been investigated in cats. The goal of this study was to assess the in vitro effects of 6 % HES 130/0.42 on feline whole blood samples using rotational thromboelastometry (ROTEM). A further goal was to develop feline reference intervals for ROTEM at our institution. In this in vitro experimental study, blood samples of 24 adult healthy cats were collected by atraumatic jugular phlebotomy following intramuscular sedation. Baseline ROTEM analyses (using ex-tem, in-tem and fib-tem assays) were performed in duplicate. Additionally, ROTEM analyses were performed on blood samples after dilution with either Ringer’s acetate (RA) or 6 % HES 130/0.42 (HES) in a 1:6 dilution (i.e. 1 part solution and 6 parts blood).

**Results:**

Coefficients of variation of duplicate measures were below 12 % in all ex-tem assays, 3 of 4 in-tem assays but only 1 of 3 fib-tem assays. Reference intervals were similar albeit somewhat narrower than those previously published. Dilution with both solutions lead to significantly prolonged CT (in-tem), CFT (ex-tem and in-tem), and reduced MCF (ex-tem, in-tem, and fib-tem) and alpha (ex-tem and in-tem). Compared to RA, dilution with HES caused a significant prolongation of CT in fib-tem (*P* = 0.016), CFT in ex-tem (*P* = 0.017) and in-tem (*P* = 0.019), as well as a reduction in MCF in in-tem (*P* = 0.032) and fib-tem (*P* = 0.020), and alpha in ex-tem (*P* = 0.014). However, only a single parameter (CFT in ex-tem) was outside of the established reference interval after dilution with HES.

**Conclusions:**

In vitro hemodilution of feline blood with RA and HES causes a small but significant impairment of whole blood coagulation, with HES leading to a significantly greater effect on coagulation than RA. Further studies are necessary to evaluate the in vivo effects and the clinical significance of these findings.

## Background

The artificial colloid, hydroxyethyl starch (HES), is recommended for the treatment of anesthesia-induced hypotension, for resuscitation and to increase colloid-osmotic pressure in cats [1, 2]. Although various doses have been recommended [3, 4], no study has thus far evaluated the side effects of HES solutions or established safe doses in cats.

Until recently, HES was widely used in human intensive care units throughout the world and was considered the artificial colloid of choice for resuscitation, especially in Europe [5]. The physicochemical properties of modern, third generation HES solutions (i.e. HES 130/0.4 and HES 130/0.42) were aimed to improve its safety profile and minimize adverse effects, such as kidney injury, tissue storage, and coagulopathy [3, 4]. However, results of recent clinical trials showed risks related to kidney dysfunction, hemorrhage, and increased mortality in septic and critically-ill human patients [6–8]. As a result, the use of HES in humans has been restricted by international authorities [9]. As little data is available with regards to safety in small animals, questions regarding the use of various HES solutions in veterinary medicine have arisen [3, 10].

The pathophysiology of hemostatic effects of HES is well described in several human and veterinary medical reviews [4, 11]. Indirect effects, due to hemodilution, as well as direct effects of HES molecules on components of the hemostatic system lead to an increased risk for hemorrhage [11–13]. Recent canine studies revealed a dose-dependent impairment of coagulation and platelet function with different HES products [14–18]. However, no data evaluating the effects of HES on feline hemostasis have thus far been published.

Assessment of whole blood coagulation using rotational thromboelastometry (ROTEM) has previously been evaluated in dogs and reference intervals have been established [19]. In addition, the effects of tetrastarch solutions on canine whole blood coagulation have been evaluated [15, 18, 20]. In cats, rare studies have been performed evaluating whole blood coagulation using thromboelastography [21–25] and recently, one study, assessing reference intervals in healthy cats using ROTEM was published [26]. To the authors’ knowledge, the effects of isotonic crystalloids or HES on feline whole blood coagulation using ROTEM have not been previously evaluated.

The objective of the present study was to assess the in vitro effects of HES 130/0.42 compared to isotonic crystalloids on ROTEM analyses in feline blood. The hypothesis was that both solutions cause impairment of whole blood coagulation due to hemodilution and that HES would impair coagulation to a greater extent than crystalloids. A secondary objective was to develop institution-specific reference intervals for ROTEM analyses in cats.

## Methods

### Cats

The study protocol was approved by University of Bern and the Animal Experiment Committee of the Swiss Federal Veterinary Office (No. 37/14). Informed owner consent was obtained before study enrollment. Thirty-four staff-owned cats with a minimum body weight of 3 kg and an age between 1 and 12 years old were initially enrolled in the study. Cats were included if they were deemed healthy based on history, clinical examination, unremarkable results of a CBC (Advia® 120, Siemens Healthcare Diagnostics AG, Zurich, Switzerland), biochemistry panel (Cobas® c501, Roche Diagnostics, Rotkreuz, Switzerland), and standard coagulation profile (STart 4 Hemostatis Analyzer ®, Stago CH SA, Zurich, Switzerland), including prothrombin, activated partial thromboplastin and thrombin times, and fibrinogen using the Clauss method, as well as negative results for feline immunodeficiency virus and feline leukemia virus ELISA tests (SNAP® Combo Plus FIV/FeLV, IDEXX Europe, Hoofddorp, Netherlands). In addition, cats were excluded if evidence of cardiomyopathy was detected using two-dimensional and M-mode echocardiographic examination, performed by a board-certified veterinary cardiologist (AK). Cats were also excluded if they had received any medication or vaccinations, or infusions with synthetic colloids or blood products within 4 weeks prior to study enrollment.

### Study fluids

The HES product used in this study was a potato-derived HES with a molecular weight of 130 kDa and a molar substitution of 0.42 in a concentration of 60 g/L (6 % solution) in an electrolyte-balanced and buffered carrier solution (Tetraspan®, BBraun Melsungen AG, Melsungen, Germany). The crystalloid was a modified Ringer’s acetate (RA) solution (Ringerfundin®, BBraun Melsungen AG, Melsungen, Germany).

### Rotational thromboelastometry

Three four-channel ROTEM (ROTEM®, TEM Innovations GmbH, Munich, Germany) analyzers were used to assess whole blood coagulation. The method dynamically analyses the viscoelastic properties of blood during initiation and propagation of clotting, as well as during fibrinolysis and is described in detail elsewhere [27]. Recalcification was performed using the calcium chloride reagent provided by the manufacturer (star-tem®, Tem International GmbH, Munich, Germany). Activating reagents used were ex-tem (ex-tem®, Tem International GmbH, Munich, Germany), which triggers the extrinsic pathway using tissue factor, in-tem (in-tem®, Tem International GmbH, Munich, Germany), which triggers contact activation using ellagic acid, and fib-tem (fib-tem®, Tem International GmbH, Munich, Germany), which triggers the extrinsic pathway with platelet inhibition using cytochalasin D. Data obtained from the ROTEM analyzers were clotting time (CT, the time from activation until the onset of clotting), clot formation time (CFT, time between the onset of clotting and clot firmness with an amplitude of 20 mm) (in-tem and ex-tem only), maximum clot firmness (MCF, maximum amplitude of the curve) and alpha (α, slope of the tangent).

### Blood sampling procedure

Cats were sedated by intramuscular injection of 0.3 mg/kg butorphanol (Morphasol® 10, Dr. E. Graeub AG, Bern, Switzerland), 2 mg/kg ketamine (Ketasol 100®, Dr. E. Graeub AG, Bern, Switzerland) and 0.2 mg/kg midazolam (Dormicum®, Roche Pharma Schweiz AG, Reinach, Switzerland). Blood was sampled by atraumatic phlebotomy of the jugular vein using a 21G butterfly needle. An initial 1.5 ml of blood was collected using a syringe attached to the butterfly needle in EDTA and heparin tubes (for CBC and biochemistry), followed by connection to a vacutainer system (BD Vacutainer® Push Button blood collection set, 21 G butterfly needle BD, Plymouth, United Kingdom) and filling of three 1.8 ml citrate tubes (BD Vacutainer® 1.8 ml coagulation tube, buffered trisodium citrate 3.2 %, BD, Plymouth, United Kingdom) for coagulation profiles and ROTEM analyses.

### Dilutions and measurements

After a resting period of 15 min, baseline ROTEM measurements to establish reference intervals were performed in duplicate. The remaining citrated blood was diluted 1:6 (1 part study fluid and 6 parts blood) with RA and HES, respectively. The appropriate amount of blood was filled in prewarmed polypropylene tubes containing either HES or RA with a pipette. Mixing was performed by careful inverting and rolling the tube 6 times, followed by 5 min’ incubation. The dilution was selected to simulate a bolus administration of 10 ml/kg, taking into account a blood volume of approximately 62–66 ml/kg body weight in an adult cat [28]. Three ROTEM devices were used in parallel and all measurements were started within 60 min after blood sampling. Samples for ROTEM measurements were stored at 37 °C in a thermostatic tube heater (WiseTerm Heating Block R48®; WITEG Labortechnik GmbH, Wertheim, Germany) and analyses were performed according to the manufacturer’s instructions. Run time was preset to 60 min. As blood collection and sample handling was shown to significantly affect whole blood coagulation in dogs [19], a single investigator (NAA) collected all samples and run all ROTEM analyses.

### Statistical analysis

Data were assessed for normality using Q-Q plots and D’Agostino-Pearson tests. As some ROTEM data were normally distributed but others were not and could not be transformed to normality, nonparametric analyses were performed. A Friedman test was used to assess differences between samples. Where significant differences were found, a post-hoc analysis was performed using Wilcoxon paired-samples tests with Bonferroni correction. The coefficient of variation of baseline duplicate measurements was calculated. Double-sided reference intervals were determined based on the average of the two repeated baseline values, using the robust method (CLSI guideline C28-A3) with confidence intervals estimated by bootstrapping. All statistical analyses were performed using commercial software (MedCalc Software® version 14.10, Ostend, Belgium) and significance was set at *P* < 0.05 throughout.

## Results

Of the 34 cats initially enrolled, ten cats were excluded due abnormal results of echocardiographic examinations (*n* = 4), anemia (*n* = 1), increased plasma creatinine concentrations (*n* = 1), prolonged prothrombin time (*n* = 1), thrombocytopenia (*n* = 1), and decreased fibrinogen levels (*n* = 1). Only data from the remaining 24 cats were included in analyses. These were domestic shorthair cats (*n* = 22) and Bengal cats (*n* = 2). The cats had a mean age of 4.5 years (range, 1.5–11.2 years) and a mean body weight of 4.5 kg (range, 3.0–6.4 kg). Twelve cats were male (1 sexually intact, 11 castrated) and 12 were female (all spayed).

### Hematologic variables

The mean (±SD) of hematologic variables included hematocrit, 31 ± 5 % (0.31 ± 0.05 L/L); platelet count, 267 ± 79 × 10^9^/L; prothrombin time, 11.2 ± 0.6 s; activated partial thromboplastin time, 12.4 ± 1.2 s; and thrombin time, 16.9 ± 1.8 s. The median (range) of fibrinogen concentrations was 143 (102–294) mg/dL.

### Reference intervals

Reference intervals were evaluated after exclusion of the two Bengal cats. These showed considerable intra-individual variation. In particular, over a four-fold difference from the lower to upper reference interval was observed for in-tem CFT and fib-tem MCF (Table 1). Coefficients of variation (CV) of duplicate measures were generally below 12 %. However, CFT in in-tem (CV, 24.2 %) and both CT (CV, 15.3 %) and MCF (CV, 20.6 %) in fib-tem showed larger imprecision (Table 2).

**Table 1:** Reference intervals for ROTEM assays in 22 cats

Assay	Interval	CT (s)	CFT (s)	MCF (mm)	Alpha (°)
ex-tem®	lower (90 % CI)	35 (31–41)	38 (34–44)	62 (59–64)	74 (73–76)
	upper (90 % CI)	71 (64–76)	78 (71–83)	74 (72–75)	82 (81–83)
in-tem®	lower (90 % CI)	107 (87–130)	23 (12–33)	52 (46–58)	70 (67–74)
	upper (90 % CI)	221 (197–244)	90 (77–101)	82 (76–86)	87 (83–90)
fib-tem®	lower (90 % CI)	38 (34–43)	n/a	5 (2–9)	52 (48–56)
	upper (90 % CI)	66 (62–70)	n/a	25 (22–24)	81 (76–85)

*90 % CI* 90 % confidence interval, *n/a* not applicable

**Table 2:** Coefficients of variation (CV) of duplicate measure for ROTEM assays in 22 cats

Assay	CV (%)			
	CT	CFT	MCF	Alpha
ex-tem®	8.95	7.96	4.72	2.08
in-tem®	11.95	24.20	3.38	2.92
fib-tem®	15.28	n/a	20.63	7.21

*n/a* not applicable

### Dilution with ringer’s acetate and HES

Significant differences were observed between baseline ROTEM values and the samples diluted with RA or HES (Table 3). The dilution with both solutions lead to significantly prolonged CT in-tem (RA, *P* = 0.0006; HES, *P* = 0.002), CFT ex-tem (RA, *P* = 0.0006; HES, *P* = 0.0003) and in-tem (RA, *P* = 0.015; HES, *P* = 0.0003), as well as to reduced MCF ex-tem (RA and HES *P* = 0.0003), in-tem (RA and HES, *P* = 0.0003) and fib-tem (RA and HES, *P* = 0.0003) and alpha ex-tem (RA, *P* = 0.0006; HES, *P* = 0.0003) and in-tem (RA, *P* = 0.017; HES, *P* = 0.0003) compared to baseline (Table 3). In addition, dilution with RA alone lead to a shortening of CT ex-tem (*P* = 0.015).

**Table 3:** ROTEM analyses in feline blood samples without dilution (baseline), and samples diluted 1:6 with RA or HES

Assay	Variable	Baseline	RA	HES
ex-tem®	CT (s)	52 (40–73)	48^*^ (31–65)	52 (31–99)
	CFT (s)	57 (44–77)	65^*^ (44–123)	↑81^*§^ (61–146)
	MCF (mm)	68 (60–74)	64^*^ (53–71)	64^*^ (56–69)
	alpha (°)	78 (75–81)	76^*^ (67–81)	74^*§^ (63–77)
in-tem®	CT (s)	165 (131–246)	203^*^ (153–303)	202^*^ (154–282)
	CFT (s)	54 (35–97)	67^*^ (46–141)	73^*§^ (50–190)
	MCF (mm)	68 (50–72)	62^*^ (47–68)	60^*§^ (42–67)
	alpha (°)	79 (72–83)	77^*^ (63–80)	76^*^ (66–80)
fib-tem®	CT (s)	53 (37–62)	49 (30–65)	60^§^ (31–93)
	MCF (mm)	15 (4–23)	9^*^ (3–16)	8^*§^ (2–14)
	alpha (°)	66 (54–77)	63 (47–75)	53 (43–62)

Values are expressed as median (range). * denotes a significant difference (*P* < 0.05) between baseline and diluted samples; § denotes a significant difference (*P* < 0.05) between samples diluted with HES and RA; ↑ denotes post-dilution median values above the established reference interval

Compared to RA, dilution with HES lead to prolongation of CT fib-tem (*P* = 0.016), CFT ex-tem (*P* = 0.017) and in-tem (*P* = 0.019), as well as reduction of MCF in-tem (*P* = 0.032) and fib-tem (*P* = 0.020), and alpha ex-tem (*P* = 0.014).

Despite statistically significant differences in ROTEM assays after dilution with study solutions, the median measured value was outside of the established reference range only after dilution with HES in ex-tem CFT (Table 3).

## Discussion

This study demonstrates that in vitro hemodilution of feline blood with RA and HES affects whole blood coagulation measured by ROTEM, with HES leading to a greater hemostatic impairment than RA. These findings corroborate those reported in humans and in dogs [11, 15, 29]. After both solutions, ROTEM parameters showed a hypocoagulable pattern, characterized by a longer duration of clot formation and propagation time (increased CFT and decreased alpha), and lower clot stability and less stable fibrin network (decreased MCF). However, the magnitude of the observed changes was low, and only a single parameter (CFT ex-tem) was outside of the reference interval established after dilution with HES, suggesting that findings may not be of clinical relevance. In a recent study, evaluating the effects of HES 130/0.4 on canine ROTEM parameters, 25 % dilution resulted in 7 of 13 parameters being outside the of reference intervals although all parameters were within reference limits after 10 % dilution [15]. In a similar study in dogs with the same HES preparation, 25 % dilution resulted in 4 out of 5, and 10 % dilution resulted in 2 out of 5 ROTEM parameters being significantly impaired compared to the dilutional control with saline [18]. Several reasons may be responsible for the smaller effects on feline coagulation in the present study. Firstly, the dilution used was 1:6 (14 %) compared to 1:3 (25 %) in the aforementioned canine studies. This likely resulted in lesser coagulation impairing effects in the present study. Moreover, feline platelets are more reactive than those of other species, due to larger platelet size and higher concentrations of serotonin [30]. Finally, the HES preparation used in the present study was a potato-derived starch in a calcium-containing carrier solution, in contrast to the waxy-maize-derived starch in calcium-free solution used in the aforementioned canine studies. This might have influenced ROTEM parameters, although only minor effects on coagulation were found when comparing calcium-containing versus calcium-free carrier HES preparations in a recent in vivo study in dogs [20]. The reason why CFT ex-tem was apparently affected more than other parameters following dilution with HES in the present study is unclear, but similar findings were observed in canine studies at low dilutions [15, 18]. Despite the overall relatively minor effects observed, data in the present study suggest that dilution with HES has a more pronounced effect on whole blood coagulation than RA, suggesting that effects of HES are beyond that of dilution alone.

Little data on ROTEM analyses in cats are currently available. Notably, no data exist regarding the association between ROTEM parameters and clinically relevant impairment of hemostasis. Previous studies found that cats with laboratory evidence of disseminated intravascular coagulation rarely show clinical bleeding tendencies [31, 32]. The observed changes in ROTEM analysis after dilution with HES may therefore not be of clinical significance at the dose simulated in the current study.

Recently, feline reference intervals for ROTEM using ex-tem and in-tem assays, based on 55 clinically healthy European shorthair cats, were published [26]. Reference ranges established in the present study were similar although a narrower interval was found for some of the parameters. However, reference intervals established in the present assay were based on a smaller population, and the robust method was used instead of a percentile method. Intra-assay coefficients of variation, evaluated from quadruplicate measurements, were found to be below 10 % for CT, CFT, alpha, and MCF in a previous study [26]. In another study, intra-assay CV was below 5 % in kaolin-activated thromboelastography in 30 healthy cats [24]. The coefficients of variation in our study were below 10 % in 7 of the 11 ROTEM parameters, but were between 10 and 25 % in another 4 parameters. However, CVs established in the present study were based on duplicate measurements in all 24 samples, but those previously published were based on quadruplicate measurements in only two samples. Moreover, although the mean of the 2 CVs was below 10 % in the previous study, the CV of one of the two samples was above 10 % in 3 assays. Lastly, evaluation of CVs using the fib-tem assay was not performed in previous studies.

Although HES-associated coagulopathy may in part be due to dilution alone, direct effects of HES on hemostatic components have been observed [4, 11]. Of these, an acquired impairment of fibrin polymerization is suggested to be most important. A sensitive parameter for monitoring fibrin polymerization with ROTEM is MCF, in particular in the fib-tem assay. In the present study, HES led to a significantly lower MCF compared to RA in both the in-tem and fib-tem assays, but the median value was still within the reference interval. Impaired platelet function is another important aspect of HES-induced coagulopathy. ROTEM parameters which are influenced by both platelet numbers and function are CFT, alpha, and MCF. A separate platelet function analysis might have added more specific information about the platelet impairing effect of HES in cats.

As previous studies have demonstrated that cardiac disease can affect coagulation in humans as well as in cats and dogs [33–36], an attempt to exclude cats with subclinical cardiomyopathy using echocardiography was made in the present study. Indeed, one former study evaluating the prevalence of cardiomyopathy in apparently healthy cats identified cardiomyopathy in 16 % of the cats [33]. Similarly, four of the originally enrolled 34 cats were excluded based on abnormal echocardiography in the present study. As echocardiography was not performed in the previous study reporting feline reference intervals, the extent to which this may explain the wider intervals previously reported is unclear. Unfortunately, urinalysis was not performed in the present study as part of the health assessment. Although plasma urea and creatinine concentrations were within reference ranges, early renal disease may have gone unnoticed. Furthermore, cats were not evaluated for hemotropic mycoplasmas or *Cytauxzoon felis* infection.

The study has some limitations. Firstly, all cats underwent sedation to ensure atraumatic jugular venipuncture and to harvest the required amount of blood for all analyses without interruption of blood flow. A previous study in cats found that ketamine may slightly increase prothrombin time and partial thromboplastin time after intravenous administration in combination with diazepam [37]. However, the observed changes were small and likely not of clinical relevance [37]. Another study showed differences in kaolin-activated thromboelastographic tracings between non-sedated cats and those sedated with butorphanol and midazolam, with the latter having a mild increase in the rapidity of clot formation [24]. Neither reaction time nor maximum amplitude was significantly affected in the sedated cats. Therefore, the authors of that study suggested that the effects on thromboelastographic variables may not be of major importance. Due to the lack of studies using ROTEM in cats, the extent to which prior sedation may have influenced results in the present study is unclear. In addition, ROTEM measurements were started after 15 min resting time at 37 °C instead of 30 min at room temperature, as recommended by the PROVETS guidelines [38]. This was done in order to perform all analyses within 60 min of blood collection and avoid temperature variations, but may have affected results in some samples. Furthermore, the effects of transendothelial fluid shifts was excluded due to the in vitro character of the study. In addition, vasoactive effects of fluids, endogenous release of fibrinolytic factors, clot dissolution, excretion or degradation of HES, and extraneous factors, such as stress response, tissue damage, and endothelial injury are not taken into account in an in vitro model. Results of the current and other in vitro studies do not therefore mimic in vivo conditions. A further limitation was the dilution of citrated blood samples with citrate-free study solutions, which may have resulted in an excess in calcium concentrations following the standardized recalcification in the ROTEM cup. This in turn could impact thrombin generation and diminished the hypocoagulable effects of HES. Finally, only healthy cats were enrolled in this study, and the effects of fluid therapy on coagulation may be more pronounced in cats with pre-existing coagulopathies.

## Conclusion

Data from the present study suggest that an in vitro 1:6 dilution with HES 130/0.42 affects feline whole blood coagulation to a greater extent than a similar dilution with RA. However, the magnitude of the observed changes was small, and only a single parameter was outside of the established reference interval after dilution with HES. Further studies are necessary to evaluate the in vivo risk of hemostasis impairment following administration of HES in healthy cats and those with naturally occurring disease and pre-existing coagulopathies.

## Abbreviations

CFT, clot formation time; CT, clotting time; ex-tem, extrinsic thromboelastometry; fib-tem, fibrinogen function thromboelastometry; HES, hydroxyethyl starch; in-tem, intrinsic thromboelastometry; MCF, maximal clot firmness; RA, Ringer’s acetate; ROTEM, rotational thromboelastometry
